# Sexual Violence Trends before and after Rollout of COVID-19 Mitigation Measures, Kenya

**DOI:** 10.3201/eid2813.220394

**Published:** 2022-12

**Authors:** Walter Ochieng, Elizabeth O’Mara Sage, Thomas Achia, Patricia Oluoch, Caroline Kambona, John Njenga, Marc Bulterys, Aun Lor

**Affiliations:** US Centers for Disease Control and Prevention, Atlanta, Georgia, USA (W. Ochieng, E. O’Mara Sage, A. Lor);; US Centers for Centers for Disease Control and Prevention, Nairobi, Kenya (T. Achia, P. Oluoch, C. Kambona, J. Njenga, M. Bulterys)

**Keywords:** COVID-19, respiratory infections, severe acute respiratory syndrome coronavirus 2, SARS-CoV-2, SARS, coronavirus disease, zoonoses, viruses, coronavirus, sexual violence, time-series analysis, trends, Kenya

## Abstract

COVID-19 mitigation measures such as curfews, lockdowns, and movement restrictions are effective in reducing the transmission of SARS-CoV-2; however, these measures can enable sexual violence. We used data from the Kenya Health Information System and different time-series approaches to model the unintended consequences of COVID-19 mitigation measures on sexual violence trends in Kenya. We found a model-dependent 73%–122% increase in reported sexual violence cases, mostly among persons 10–17 years of age, translating to 35,688 excess sexual violence cases above what would have been expected in the absence of COVID-19–related restrictions. In addition, during lockdown, the percentage of reported rape survivors receiving recommended HIV PEP decreased from 61% to 51% and STI treatment from 72% to 61%. Sexual violence mitigation measures might include establishing comprehensive national sexual violence surveillance systems, enhancing prevention efforts during school closures, and maintaining access to essential comprehensive services for all ages and sexes.

COVID-19 mitigation measures such as curfews, lockdowns, and travel restrictions reduce disease transmission, but these measures also disrupt economic activities and social networks, and hinder access to health and social services (*1*,*2*). Mass disruption of socioeconomic activities often has unintended consequences, including an increase in sexual violence and prolonged exposure to abusers, while concomitantly limiting survivors’ access to and the availability of medical and social services (*2*–*5*).

A COVID-19 case was confirmed in Kenya on March 13, 2020. The government rolled out a series of measures to contain the spread of COVID-19 and mitigate its impacts on March 15, 2020. These measures included school closures, movement restrictions, curfews, rescheduling of clinical services, and reassignments of health workers to COVID-19 case management (Appendix Figure 1).

In May 2020, the United Nations Population Fund warned that an additional 31 million cases of sexual and gender-based violence would be seen globally during implementation of COVID-19 mitigation measures and called on governments to be alert to these dangers (*6*). In July 2020, one study found that patterns of sexual violence against children in Kenya had changed and that the average age of survivors declined from 16 to 12 years (H.D. Flowe et al., unpub. data, https://doi.org/10.31234/osf.io/eafwu). That study also found that 76% of offenses occurred during the day and coincided with normal school hours. Another study during the lockdown noted that 78% of perpetrators were known to the victim, either family members or neighbors (*7*). These studies were not designed to quantify national estimates of sexual violence, but they attest to the heightened exposure of women and girls to sexual violence.

To determine whether sexual violence increased in Kenya during the COVID-19 pandemic, we examined trends in reported sexual violence cases in Kenya during January 2015–June 2021. Because COVID-19 mitigation measures also disrupted clinical services, we assessed changes in overall quality of care for sexual violence survivors, including HIV postexposure prophylaxis (PEP) and sexually transmitted infection (STI) treatments. 

## Methods

### Definitions and Data Sources

We obtained monthly sexual violence reports from the Kenya District Health Information System (DHIS2) database covering January 2015–June 2021. Those data cover patients who received clinical care in hospitals, health centers, and dispensaries registered by the Kenya Medical Practitioners and Dentists board. Those health facilities also offer other routine clinical services, such as malaria treatment. Aggregate facility-level sexual violence data are extracted from the Kenya Ministry of Health Sexual Gender-Based Violence (SGBV) register (MOH 365) and entered into DHIS2 monthly; patient-level data are not available in the DHIS2 database. 

We used sexual violence case definitions as outlined in the National Guidelines on the Management of Sexual Violence in Kenya (*8*) and the SGBV register (*9*). Those documents outline acts of sexual violence list acts of sexual violence as rape, attempted rape, defilement, incest, sexual assault, gang rape, and forcible anal penetration (*8*). Rape covers forcible anal penetration in both sexes (*8*). In contrast, the legal definition of rape in Kenya is forcible vaginal penetration only.

We appraised the following data: overall sexual violence, a general category that includes attempted rape and other unspecified forms or types of sexual violence; rape, including forcible penetration of vagina or anus; rape-related HIV PEP; and rape-related STI treatment. We included PEP and STI treatment outcomes to assess whether the sexual violence survivors received minimum standard care according to the national guidelines (*8*) or if standards of care changed during the pandemic. Because we expected these 2 indicators to directly correlate, they also served as data quality checks for overall sexual violence and rape cases.

Of note, a registered facility can report a sexual violence survivor as a sexual violence or rape case and document whether the patient received HIV PEP or STI treatment. At the end of each month, the facility aggregates reports for and enters information into DHIS2 using inputs for the total number of rape consultations (rape), among which the facility notes the number of rape survivors who received PEP (rape-PEP), and the number who received STI treatment (rape-STI). Because dispensaries and health centers might collect information in paper or electronic form before data are entered into DHIS2, 1 sexual violence survivor might be reported to DHIS2 <3 times, as rape, rape-PEP, or rape-STI. 

**Figure 1:**
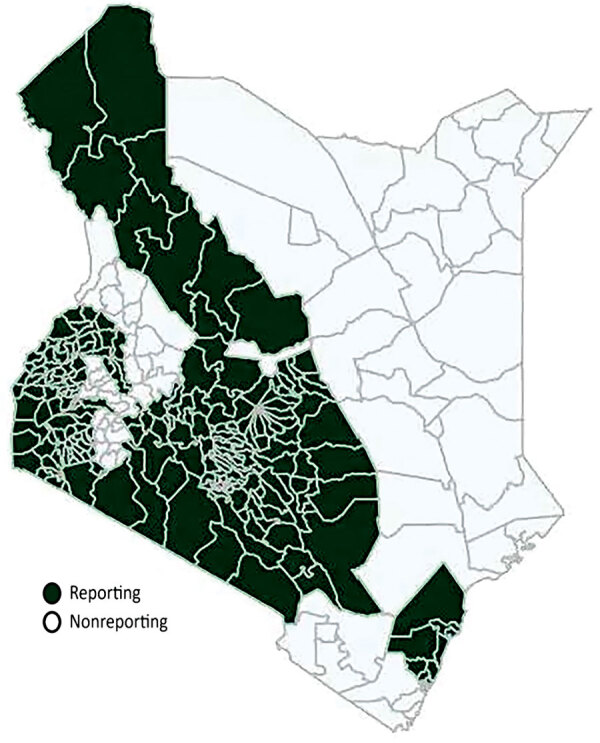
Counties reporting sexual violence cases before and after rollout of COVID-19 mitigation measures, Kenya, January 2015–June 2021. The shaded areas indicated counties that have complete sexual violence reports in the Kenya DHIS-2 database, which were included in the analyses. The following counties did not report sexual violence data to the DHIS-2: Baringo, Bomet, Elgeyo-Marakwet, Garissa, Isiolo, Kericho, Kwale, Lamu, Mandera, Marsabit, Nandi, Taita Taveta, Tana River, Wajir, and West Pokot. DHIS-2, District Health Information System 2.

Of 47 counties in Kenya, we excluded 14 (30%) from our analysis because they had incomplete or missing data in DHIS2. The excluded counties account for ≈19.5% of the country’s population (Figure 1).

### Statistical Approaches and Assumptions

We hypothesized that monthly reported cases of sexual violence evolve with time, based on changing sociocultural, policy, and legal factors. We also hypothesized that seasonal variations in sexual violence occur and that case numbers would be higher in some months than others; thus, we hypothesized both long-term trends and seasonal patterns in reported sexual violence cases. To calculate the effect of COVID-19 lockdowns on sexual violence, we followed the traditional time-series approach and estimated the trends that would have been expected during the lockdown, had the lockdown not happened. We considered the difference between the reported cases and the estimated nonlockdown trend as effects of the lockdown.

We first conducted descriptive analyses and checked for seasonal patterns in sexual violence by separating monthly variations from long-term trends (Appendix Figure 2). We then conducted several statistical tests to select the most appropriate time-series models (Appendix). We used those selected models to estimate the effects of the lockdowns on sexual violence and quality of sexual violence survivor care.

We made several assumptions for our analyses to make our models realistic. First, we assumed no changes in data reporting occurred during the study period, including changes in reporting requirements, definitions of indicators, or data collection tools. We checked this assumption by examining data quality reports from the DHIS2 database and through discussions with public health program officers working on sexual violence in Kenya.

Second, we assumed that no factors or events that could affect sexual violence trends, but were unrelated to the pandemic, were occurring when the lockdown started. Such factors might include new legislation penalizing sexual violence or mass disruptive events, such as civil conflict. We checked this assumption through discussions with program staff, by using date falsification tests to change the lockdown start date to several months before and after March 2020, and by using Supremum Wald tests to look for unusual patterns in the data (*10*).

**Figure 2:**
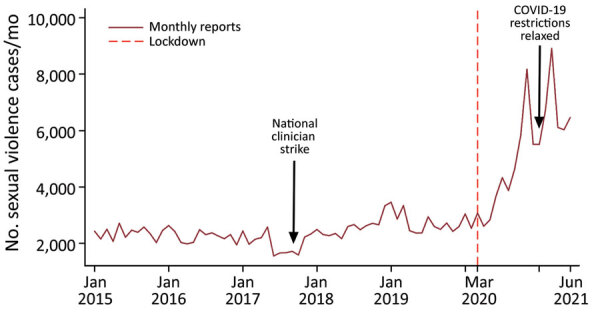
Overall unadjusted trends in sexual violence cases before and after rollout of COVID-19 mitigation measures, Kenya, January 2015–June 2021. The graph shows monthly number of reported sexual violence cases; vertical red dashed line represents the official start of the COVID-19 pandemic and associated lockdowns in Kenya.

Third, we assumed that potential perpetrators at the population level were unaware of impending lockdowns and subsequently did not modify their behavior in anticipation of the lockdown. Any prelockdown anticipatory effects would have biased the nonlockdown estimates upward or downward depending on the direction of the effects (*11*). We tested this assumption by using date falsification and Supremum Wald tests and by examining raw sexual violence trend graphs (Figure 2; Appendix). Because time-series analyses require >50 observations for stable estimates of the underlying trend and to model for seasonality, we expanded our dataset to include 78 observation months (*12*,*13*).

Because different time-series approaches have inherent strengths and limitations, we compared estimates across different models to increase result confidence. For example, before and after analyses, we assumed no long-term trends were occurring. However, interrupted time-series require multiple observations; thus, we checked estimates of the seasonal autoregressive integrated moving average model (SARIMA) as our primary method and crosschecked the estimates by using 4 additional methods: seasonal Holt-Winters, Bayesian structured time-series (BSTS), ordinary least squares interrupted time-series analysis (ITSA), and negative binomial interrupted time-series regressions (NBREG) (Appendix).

### Software and Ethics Approval

We conducted analyses in Python version 3.7 (Python Software Foundation, https://www.python.org) and Stata version 14 (StataCorp LLC, https://www.stata.com). We developed a web-based application, SGBV Rapid Trend Analysis Tool (https://sgbv-app.herokuapp.com), for researchers who wish to conduct similar analyses. The details of the statistical methods, tests, and interpretation of results are included as part of the tool. This study was reviewed in accordance with US Centers for Disease Control and Prevention human subjects review procedures and was determined to not meet the definition of research as defined in 45 CFR §46.102(l).

## Results

We found that reported cases of sexual violence in Kenya doubled during the COVID-19 pandemic. The pre–COVID-19 (January 2015–March 15, 2020) monthly mean number of cases was 2,387 (95% CI 2,289–2,485) but rose to a monthly mean of 5,269 (95% CI 4,289–6,250) after COVID-19 lockdowns began on March 15, 2020 (Table; Figure 2). From the prelockdown to postlockdown periods, DHIS2 data inputs for rape increased from 1,037 to 1,801/month, rape-PEP increased from 628 to 910/month, and rape-STI increased from 745 to 1,115/month.

**Table:** Summary statistics of sexual violence trends before and after rollout of COVID-19 mitigation measures, Kenya*

Indicator	Prelockdown, mean (95% CI)	Postlockdown, mean (95% CI)	SARIMA parameters
Total sexual violence cases	2,387 (2,289–2,485)	5,269 (4,289–6,250)	(4,1,0) x (1,1,0,12)
Rape	1,037 (989–1,085)	1,801 (1,576–2,028)	(0,1,0) x (1,0,0,12)
Rape-PEP	628 (603–653)	910 (814–1,007)	(1,1,1)
Rape-STI treatment	745 (714–776)	1,115 (980–1,249)	(0,1,0)

*Trends were measured during January 2015–June 2020. PEP, postexposure prophylaxis for HIV; SARIMA, seasonal autoregressive integrated moving average; STI, sexually transmitted infection.

We noted a dip in the upward trajectory of reported sexual violence cases after COVID-19 restrictions were relaxed during November 2020–February 2021 (Table; Figure 2). However, a fresh upsurge in cases occurred after COVID-19 restrictions were reimposed in March 2021 (Figure 2; Appendix Figure 2).

We found that reported sexual violence cases decreased during a series of national healthcare worker strikes in 2017 (Figure 2). We also found seasonal variations in reported sexual violence cases, and that peaks typically occur during November–January, coinciding with the main school vacation in Kenya (Appendix Figure 2).

The base SARIMA model showed that, after COVID-19 mitigation measures were introduced in March 2020, reported sexual violence cases increased by a monthly average of 73% (2,229). SARIMA model estimates were more conservative than estimates using the alternate models; ITSA showed a 95% increase, NBREG 122%, and BSTS 112% (Appendix Figure 3). Those results translate to a cumulative increase of 35,668 (95% CI 28,972–42,364) reported sexual violence cases compared with the modeled scenario without COVID-19 restrictions.

**Figure 3:**
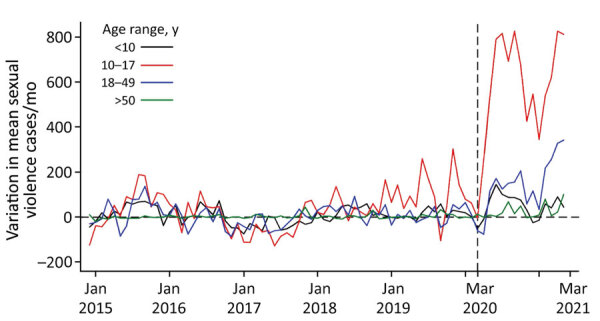
Mean sexual violence cases by age before and after rollout of COVID-19 mitigation measures, Kenya, January 2015–June 2021. Changes in age-disaggregated cases were calculated by using a Bayesian structural time series model. The horizontal dashed line represents the baseline; the vertical dashed line represents the official start of the COVID-19 pandemic and associated lockdowns in Kenya.

Overall, reported sexual violence cases increased for all age groups during lockdown, but the highest increase occurred among persons in the 10–17-year age group, which had an 117.2% increase. Other age groups also had increased rates: 20.7% among persons <10 years of age, 37.2% among those 18–49 years of age, and 16.3% among those >50 years of age (Figure 3; Appendix Figure 4). 

We found a model-dependent 22%–76% increase in monthly reported rape cases (Appendix Table). The proportion of rape survivors receiving the minimum package of standard care recommended by national guidelines (*8*) declined. Of note, during the prelockdown period, only 61% of rape cases were reported to have received PEP, and only 72% received STI treatment. In the postlockdown period, the proportion of rape survivors receiving PEP declined from 61% to 51%, and those receiving STI treatments declined from 72% to 61%; however, the number of PEP and STI treatments administered increased overall (Table).

## Discussion

During the lockdown period, we found a 73%–122% increase in reported sexual violence cases, confirming previous studies and media commentaries about an increase in sexual violence during the pandemic (*7*; H.D. Flowe et al.). Monthly reported cases increased as the lockdown progressed, and reports during December 2020 were 4 times higher than the pre–COVID-19 monthly average. Case reports moderately declined in January 2021, coinciding with relaxation of some COVID-19 mitigation measures, and surged again in March 2021 after mitigation measures were reintroduced (Figure 2; Appendix Figure 2).

During COVID-19 lockdown, reported sexual violence cases more than doubled among persons 10–17 years of age, but all age groups had increased rates (Appendix Figure 4). We hypothesize that the spike in cases among the adolescent group resulted from extended school closures, which led to increased contact time with potential abusers. Other studies using survivor-level data have shown a shift in abuse patterns to daylight hours and a decline in mean age of sexual violence survivors from 16 to 12 years of age (*7*; H.D. Flowe et al.). We were not able to assess this change with the available data.

For the period before the pandemic, our descriptive analyses found a strong seasonal pattern in sexual violence, and peaks coincided with school vacations (Appendix Figure 2). We did not find any literature regarding seasonal variation in sexual violence reports in East Africa, but program managers should consider incorporating these variations in their sexual violence intervention plans.

We found a correlated increase in 2 national indicators of the quality of sexual violence care, rape-related PEP treatment and facility-reported cases associated with STI treatment. These indicators showed an absolute increase in treatments administered (Appendix Table), but the average proportion of reported survivors receiving the minimum standard-of-care declined from 61% to 51% for PEP and 72% to 61% for STI treatment. Further studies are needed to determine why only 61% of rape cases received PEP and only 72% received STI treatment before the lockdown and why the percentage of rape cases receiving PEP and STI treatment decreased further during lockdown.

Our results mirror previous studies that found an increase in cases of sexual violence during pandemics or in the aftermath of major disasters (*1*). Our results are higher than those found in a preanalysis and postanalysis conducted by the United Nations Population Fund, which compared data from Mali in April 2019 to data from April 2020 (*14*). That analysis found a 35% increase in gender-based violence in Mali; however, the number of reporting organizations decreased from 32 to 13 during the analysis period, so these data are likely underestimates (*14*).

Our results are also consistent with a study examining patterns of sexual violence against adults and children in Kenya during the lockdown (*7*). That study found that children were more likely than adults to be victimized, primarily resulting from school closures because violations occurred more frequently during the day, by someone known to the survivor, and in private rather than a public location (*7*).

We used 4 different time-series approaches, each with their own strengths and weakness, to assess the robustness of the findings (Appendix Figure 3). We conducted several falsification and statistical tests to assess whether other competing events might have affected the results. We also assessed seasonality and secular trends, thereby avoiding biases in preanalysis and postanalysis evaluations when comparing observations from corresponding months across different years.

Our investigation likely underestimated sexual violence cases during lockdown. First, sexual violence is often underreported because of stigma, fear of retribution, cultural normalization of sexual violence, mistrust of authorities, lack of knowledge about services, and weak legal systems (*5**,**7**,**15*). Second, DHIS2 data are restricted to registered facilities, are often incomplete, and do not capture medical care received elsewhere, such as in nonregistered facilities like clinics in slums or at home. Third, because DHIS2 does not receive data from stand-alone rape crisis centers and does not receive reports from 30% of the counties in Kenya (Figure 1), especially those in North-Eastern and central Rift Valley Provinces, the DHIS2 rape data might not fully represent the total population of rape survivors in Kenya. Fourth, movement restrictions could have hindered access to medicolegal care (facilities and police). Fifth, survivors could have avoided seeking help in health facilities during the early phases of the pandemic because of fear of getting infected with SARS-CoV-2. Therefore, survivors who went to healthcare facilities during the COVID-19 pandemic could have had more severe injuries, might represent a subset of the population that could navigate pandemic restrictions such as curfews, have been of higher socioeconomic status, or lived in proximity to health facilities. We have no way of testing for this information in the data. Finally, case-patient sex was not reported to DHIS2, and we do not know how many facilities or standalone rape crisis centers provide sexual violence services to male survivors or if the sex distribution of rape cases changed during lockdown. Thus, we do not know if gaps in PEP and STI treatment were worse in male versus female sexual violence survivors.

Because we did not have patient-level data, we were unable to conduct detailed subanalyses, such as age-sex disaggregation, incident time of day or day of week, or perpetrators’ ages or their relationships with the survivors. National-level aggregates smoothed out random variations in healthcare service access and reporting at healthcare facility–level, these aggregates do not capture geographic heterogeneity in sexual violence patterns that enable more targeted interventions. Additional analyses are therefore essential.

## Conclusions

We used DHIS2 data to examine trends in reported sexual violence cases during the COVID-19 pandemic in Kenya. We found that reported sexual violence and rape cases nearly doubled during COVID-19 lockdown periods, particularly among persons 10–17 years of age. We found strong seasonal patterns in sexual violence reports before the COVID-19 pandemic, and reports spiked during school vacations.

We found that gaps in PEP and STI treatment administered to rape survivors existed in Kenya before COVID-19 lockdowns began. However, the percentage of rape survivors receiving PEP and STI treatment dropped further during the lockdown. Additional studies could investigate why gaps in PEP and STI treatment occurred. 

Nonetheless, our findings likely underestimate sexual violence in Kenya during the COVID-19 pandemic. We suggest that sexual violence surveillance systems be strengthened and expanded to include all counties in Kenya. In addition, communities could identify safe spaces for children when schools are not in session and keep safe houses open and accessible for persons fleeing abusers during lockdowns. Further studies are needed to monitor the possible additional adverse effects of COVID-19 pandemic lockdowns, such as increases in teenage pregnancies and increased incidence of HIV and STIs in children and adolescence. Because the immediate and long-term deleterious effects of sexual violence on survivors and society are unclear, additional studies to generate better quality data and policies would be useful. 

In conclusion, our findings can inform planning for future pandemics or other events that result in the mass disruption of socioeconomic activities, such as earthquakes and hurricanes (*1*). Lockdown plans and policies should include sexual violence prevention and mitigation strategies. Communities should maintain access to comprehensive sexual violence care according to national standards as an essential service for all ages and sexes during pandemic lockdowns, disasters, and national emergencies. 

Appendix Additional information on sexual violence trends before and after the COVID-19 pandemic, Kenya.
