# Supplementary material for: Sexual Violence Trends before and after Rollout of COVID-19 Mitigation Measures, Kenya
Source: Emerg Infect Dis. 2022 Dec;28(Suppl 1):S270–6. doi: 10.3201/eid2813.220394 (PMC9745229; doi:10.3201/eid2813.220394)
Supplement: Appendix — Additional information on sexual violence trends before and after the COVID-19 pandemic, Kenya. [file 22-0394-Techapp-s1.pdf]

Article DOI: https://doi.org/10.3201/eid2813.220394

# Sexual Violence Trends before and after the COVID-19 Pandemic, Kenya

## Appendix

### Statistical Analysis

We first conducted descriptive analyses. We assessed distributions and outliers for each indicator, decomposed the data to check for seasonality, secular trends, and random noise, and assessed the data for autocorrelation and partial autocorrelation. Next, we conducted unit root tests to estimate the number of lags required to make the data stationary.

Traditional quasi-experimental policy evaluation methods like difference-in-differences are unsuitable in the current scenario due to the lack of appropriate control groups. We, therefore, used time series approaches to compare sexual violence trends before and after the introduction of COVID-19 mitigation measures in Kenya on March 15, 2020.

Akin to difference-in-differences, time series methods assume that pre-policy trends, including seasonal variations and levels would remain unchanged in the post-policy period under a nonintervention counterfactual state. The estimated policy impact is therefore the difference between the counterfactual-state estimates and observed data. The validity of this approach hinges on accounting for any concurrent shocks that could affect these trends and levels, such as changes in concomitant policies, measurement processes or population composition (*1*).

Our models are based on the following assumptions. First, that there were no changes in data reporting during the pandemic. We check this assumption by examining data quality reports and through discussions with key public health program officials working on sexual violence in Kenya. Second, we assumed that there were no other concurrent events, other than the pandemic policy shock, that could drive the results. These competing events could include new legislation penalizing sexual violence or mass disruptive events like civil conflicts. We check this assumption using date falsification tests (changing the policy start dates several months before

and after March 2020), Supremum Wald tests for unknown structural breaks, and Wald tests for known structural breaks in the data (*2–4*).

Third, we assume that there were no anticipatory (i.e., Ashenfelter-type) pre-policy effects; that is, perpetrators could not adjust their behavior in anticipation of the lockdown policy because the shutdown date was driven by unanticipated global factors. Existence of prelockdown anticipatory effects would bias the estimation of counterfactual trends. We checked this assumption in part by tests outlined under the second assumption and by examining raw trend graphs around the time of policy change (lockdown); excess bunching suggested such behavior (Figure 2).

Different time series approaches have their inherent strengths and limitations. We compared estimates across different models to increase confidence with our results. We used a seasonal autoregressive integrated moving average model (SARIMA) as our base model and crosschecked the estimates by using seasonal Holt-Winters, Bayesian structural time series (BSTS), interrupted time series analysis (ITSA), and negative binomial interrupted time series regressions (NBREG).

We used a forecasting approach for the SARIMA model. Having defined the number of appropriate lags to stabilize the data by using the steps described above, we selected the most appropriate SARIMA model by using Akaike information criterion and the Ljung-Box (Q) test (*5*). We then stabilized the model by using regular and seasonal differencing and rechecked for stationarity by using augmented Dickey Fuller (ADF) unit root tests.

We introduced an additional preprocessing step to confirm if the selected SARIMA model was appropriate. We split the data into training and testing datasets as follows: training was January 2015–July 2019, and testing was July 2019–February 2020. We then compared the SARIMA forecasts against the actual observed values in the testing dataset. We evaluated forecasting performance by using root mean square errors (RMSE) and mean absolute errors (MAE), and chose the appropriate autoregression orders, trend differences, and moving average orders. We then used the dataset through February 2020 to forecast for values through June 2021 and compared forecast estimates with the actual observed values. The difference between the forecasted and actual values represents the policy impact.

The main ITSA model was specified as follows (*1*):

$$Y_t = \beta_0 + \beta_1 T_t + \beta_2 X_t + \beta_3 X_t T_t + \beta_4 M_t + \varepsilon_t$$

$$\varepsilon_t = \eta_{t-k} + z_t$$

Where $Y_t$ is an aggregated regressor (e.g., OPD visits) that is measured at equal monthly intervals $t$, $X_t$ is a 0–1 indicator variable representing the COVID-19-related lockdown in March 2020, $T_t$ is the time in months since January 2015 and $M_t$ are dummy variables for months to account for seasonality. $B_0$ is the intercept term, $\beta_1$ is the pre-lockdown slope, $\beta_2$ estimates the lockdown policy shock level change, and $\beta_3$ estimates the long-term effect of the policy change (*1*,*6*). The error term, $\varepsilon_t$ uses Newey-West standard errors to account for serial correlation (*1*,*7*).

## Assumptions and Additional Robustness Checks

Kenya experienced a series of nationwide healthcare worker strikes in 2016 and 2017 that disrupted health services (*8*). There is a risk of obtaining spurious results if the effects of these strikes were significant and sustained. We, therefore, conducted additional robustness checks with an additional dummy variable (second interruption) for the onset of the strikes in the segmented regression models. We visually inspected the decomposed data for the strike period to determine if the trends were deterministic (recovered long-term trajectories after the strike ended) or stochastic (maintained a new trend after the strike ended).

The Wald and Supremum Wald tests identified 1 significant change (structural break) in trends in 2017 coinciding with the national health worker strikes. The inclusion of this break in the models did not change the results. Our results were also robust to date falsification tests with no impacts seen when the lockdown start date was varied by several months before and after March 2020.

### Software

We performed the initial data manipulations using Python version 3.7 (Python Software Foundation, https://www.python.org). All analyses and visualizations were done by using Stata version 14.2 (StataCorp LLC, https://www.stata.com) (*9*)

**Ethical Approval**

This activity was reviewed in accordance with Centers for Disease Control and Prevention human subjects review procedures and was determined to not meet the definition of research as defined in 45 CFR §46.102(1).

**References**

1. Linden A. Conducting interrupted time-series analysis for single- and multiple-group comparisons. Stata J. 2015;15:480–500. https://doi.org/10.1177/1536867X1501500208

2. Perron P. Dealing with structural breaks. In: Mills TC, Patterson K, editors. Plagrave handbook for econometrics: econometric theory. Camden (UK): Palgrave MacMillan; 2006. p. 278–352.

3. Quandt R. Tests of the hypothesis that a linear regression system obeys two separate regimes. J Am Stat Assoc. 1960;55:324–30. https://doi.org/10.1080/01621459.1960.10482067

4. Chow G. Tests of equality between sets of coefficients in two linear regressions. Econometrica. 1960;28:591–605. https://doi.org/10.2307/1910133

5. Ljung GM, Box GEP. On a measure of lack of fit in time series models. Biometrika. 1978;65:297–303. https://doi.org/10.1093/biomet/65.2.297

6. Linden A, Adams JL. Applying a propensity score-based weighting model to interrupted time series data: improving causal inference in programme evaluation. J Eval Clin Pract. 2011;17:1231–8. PubMed https://doi.org/10.1111/j.1365-2753.2010.01504.x

7. Cumby RE, Huizinga J. Testing the autocorrelation structure of disturbances in ordinary least squares and instrumental variables regressions. Econometrica. 1992;60:185–95. https://doi.org/10.2307/2951684

8. Adam MB, Muma S, Modi JA, Steere M, Cook N, Ellis W, et al. Paediatric and obstetric outcomes at a faith-based hospital during the 100-day public sector physician strike in Kenya. BMJ Glob Health. 2018;3:e000665. PubMed https://doi.org/10.1136/bmjgh-2017-000665

9. van Rossum G, Drake FL. Python 3 reference manual. Scotts Valley (CA): CreateSpace; 2009.

**Appendix Table.** Modeled monthly change in case counts in a study of sexual violence trends before and after the COVID-19 pandemic, Kenya, January 2015–June 2021*

| Indicator | SARIMA | Negative binomial | BSTS | ITSA | Cumulative SARIMA |
|---|---|---|---|---|---|
| Sexual violence cases | | | | | |
| Total | 2,229 (73.1)† | 2,904 (121.9)† | 2,710 (113.0)† | 2,570 (95.2)† | 35,668† |
| Range | 1,337–3,121 | 1,932–3,877 | 2,488–2,930 | 1,808–3,331 | 28,973–42,364 |
| Rape cases | | | | | |
| Total | 335 (22.3)† | 779 (76.2)† | 731 (70.1)† | 545 (43.3)† | 8,714† |
| Range | 136–535 | 560–999 | 635–838 | 420–669 | 7,780–9,650 |
| Rape PEP | | | | | |
| Total cases | 123 (15.7)† | 290 (46.7)† | 267 (42.4)† | 253 (38.4)† | 1,978† |
| Range | 34–213 | 200–380 | 213–320 | 222–284 | 1,307–2,649 |
| Rape STI treatment | | | | | |
| Total cases | 125 (0.1) | 380 (51.7)† | 358 (48.2)† | 369 (49.4)† | 1,999† |
| Range | 0–247 | 258–502 | 290–427 | 310–427 | 1,059–2,939 |

*Values represent no. (% standard error). BSTS, Bayesian structural time series; ITSA, interrupted time series analysis using ordinary least squares; PEP, post-exposure prophylaxis for HIV; SARIMA , seasonal autoregressive integrated moving average; STI, sexually transmitted infection.
†p<0.01.

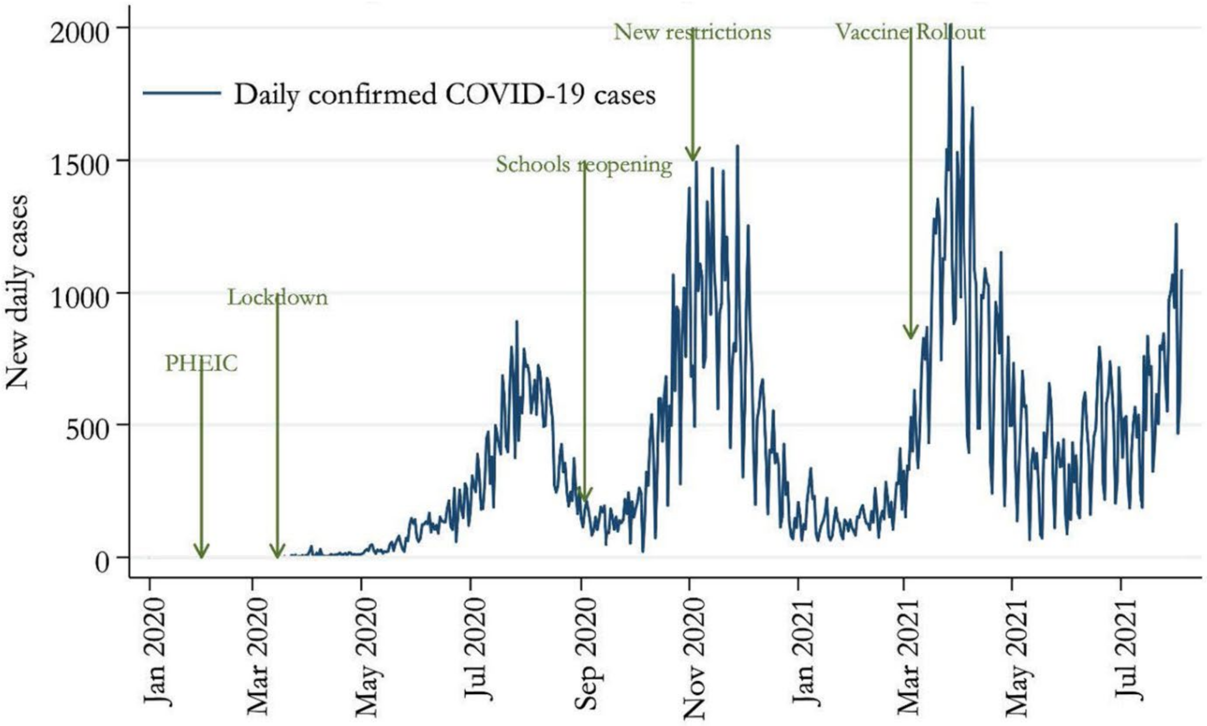


**Appendix Figure 1.** Key COVID-19 milestones used in a study of sexual violence cases before and after the COVID-19 pandemic, Kenya.

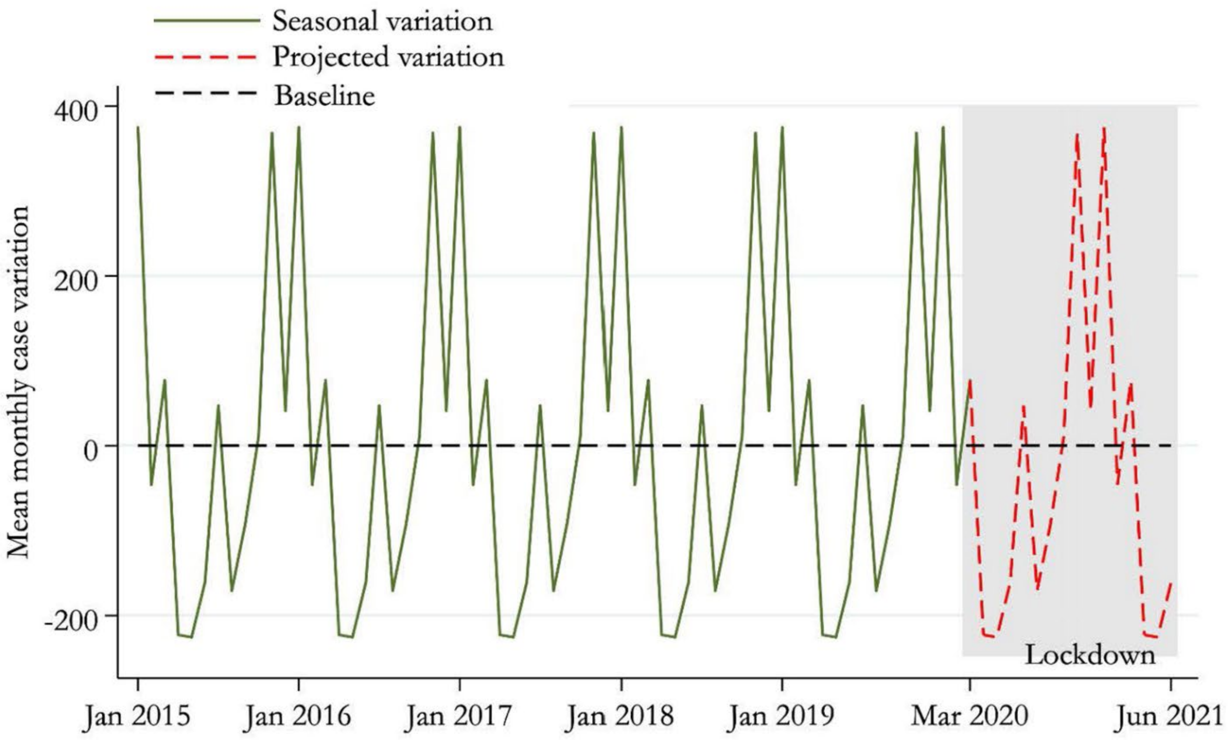


**Appendix Figure 2.** Mean seasonal variations in reported sexual violence cases before and after the COVID-19 pandemic, Kenya, January 2015–June 2021. The decomposition was performed by using an unobserved components model. The graph shows that seasonality patterns persisted during the COVID-19 lockdown period, March 2020–June 2021.

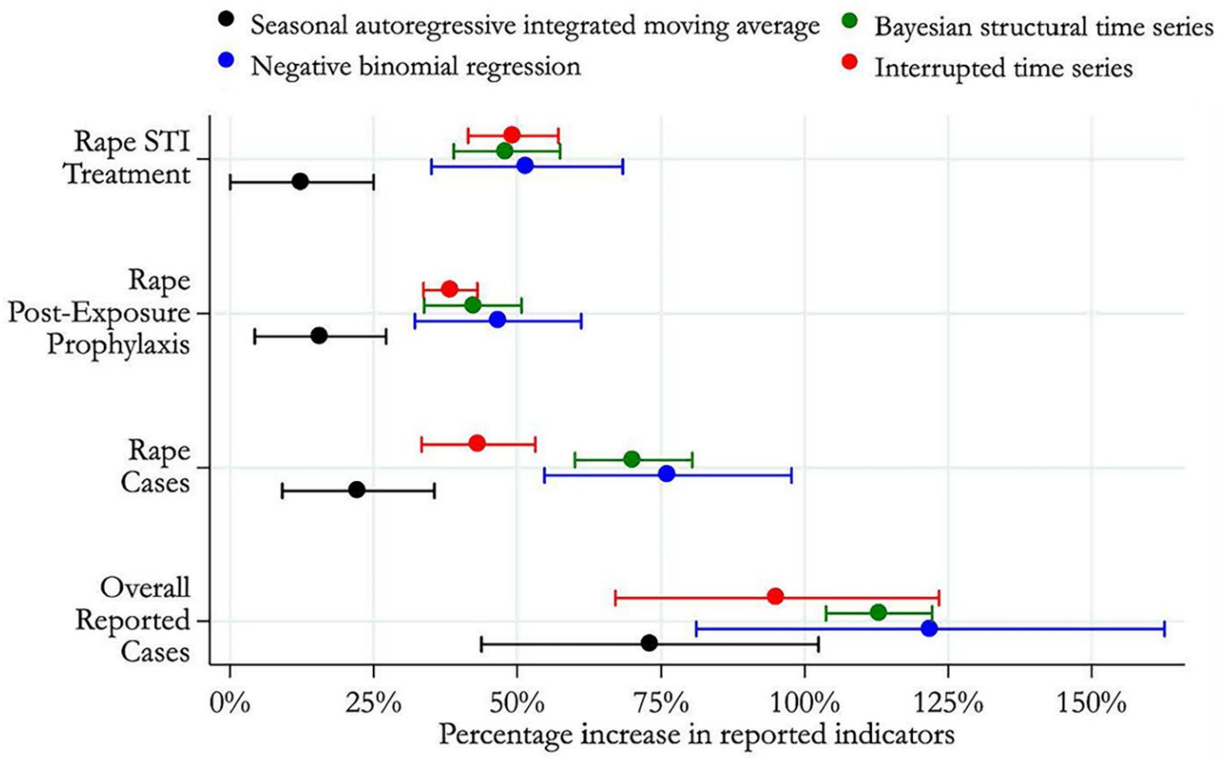


**Appendix Figure 3.** Percentage changes in reported overall sexual violence, rape, rape-related post-exposure prophylaxis, and STI treatment for rape during the COVID-19 pandemic, Kenya. Whisker plots compare seasonal autoregressive integrated moving average (SARIMA), negative binomial regression (NBREG), Bayesian structural time series (BSTS), and interrupted time series analysis (ITSA) approaches. Bars indicate range; dots indicate mean. All models show an increase above the baseline (0%), but SARIMA is most conservative and NBREG gives the largest estimates and has the widest confidence intervals. STI, sexually transmitted infection.

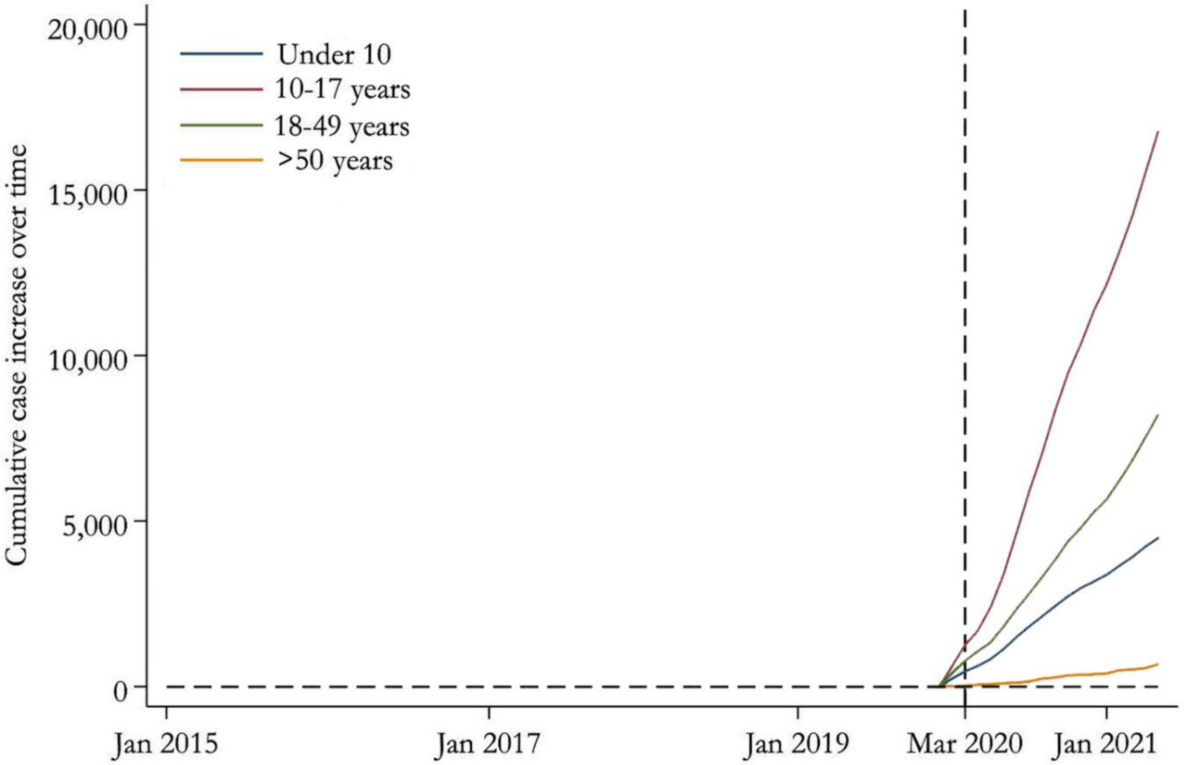


**Appendix Figure 4.** Age-disaggregated cumulative increase in sexual violence cases during the COVID-19 pandemic, Kenya. We used a Bayesian structural time series approach to assess trends. Dotted vertical line indicates the official start of the COVID-19 pandemic and associated lockdowns in Kenya.
